# Association of symptomatic upper respiratory tract infections with the alteration of the oropharyngeal microbiome in a cohort of school children in Côte d’Ivoire

**DOI:** 10.3389/fmicb.2024.1412923

**Published:** 2024-06-27

**Authors:** Kouassi Firmin Missa, Kanny Diallo, Kouakou Brice Bla, Kolotioloman Jérémie Tuo, Kossia Debia Thérèse Gboko, Laurent-Simon Tiémélé, Allassane Foungoye Ouattara, Biego Guillaume Gragnon, Joyce Mwongeli Ngoi, Robert J. Wilkinson, Gordon A. Awandare, Bassirou Bonfoh

**Affiliations:** ^1^Direction de la Recherche et du Développement, Centre Suisse de Recherches Scientifiques en Côte d’Ivoire, Abidjan, Côte d’Ivoire; ^2^Laboratoire de Biologie et Santé, UFR Biosciences, Université Félix Houphouët Boigny de Cocody, Abidjan, Côte d’Ivoire; ^3^West African Centre for Cell Biology of Infectious Pathogens, Accra, Ghana; ^4^Laboratoire de Microbiologie, Biotechnologies et Bio-informatique, Institut National Polytechnique Félix Houphouët-Boigny, Yamoussoukro, Côte d’Ivoire; ^5^Laboratoire de Cytologie et Biologie Animale, Université Nangui Abrogoua, Abidjan, Côte d’Ivoire; ^6^Laboratoire National d’Appui au Développement Agricole, Laboratoire Régional de Korhogo, Korhogo, Côte d’Ivoire; ^7^The Francis Crick Institute, London, United Kingdom; ^8^Department of Infectious Disease, Imperial College London, London, United Kingdom; ^9^Centre for Infectious Diseases Research in Africa, Institute of Infectious Disease and Molecular Medicine and Department of Medicine, University of Cape Town, Cape Town, South Africa

**Keywords:** oropharyngeal microbiome, upper respiratory tract infections, SARS-CoV-2, 16S rRNA metagenomic, microbial markers, Côte d’Ivoire

## Abstract

**Introduction:**

The oropharyngeal microbiome plays an important role in protection against infectious agents when in balance. Despite use of vaccines and antibiotic therapy to prevent respiratory tract infections, they remain one of the major causes of mortality and morbidity in Low- and middle-income countries. Hence the need to explore other approaches to prevention by identifying microbial biomarkers that could be leveraged to modify the microbiota in order to enhance protection against pathogenic bacteria. The aim of this study was to analyze the oropharyngeal microbiome (OPM) of schoolchildren in Côte d’Ivoire presenting symptoms of upper respiratory tract infections (URTI) for better prevention strategy.

**Methods:**

Primary schools’ children in Korhogo (*n* = 37) and Abidjan (*n* = 39) were followed for six months with monthly oropharyngeal sampling. Clinical diagnostic of URT infection was performed and nucleic acid extracted from oropharyngeal swabs were used for 16S rRNA metagenomic analysis and RT-PCR.

**Results:**

The clinical examination of children’s throat in Abidjan and Korhogo identified respectively 17 (43.59%) and 15 (40.54%) participants with visible symptoms of URTIs, with 26 episodes of infection in Abidjan and 24 in Korhogo. Carriage of *Haemophilus influenzae* (12%), *Streptococcus pneumoniae* (6%) and SARS-CoV-2 (6%) was confirmed by PCR. A significant difference in alpha diversity was found between children colonized by *S. pneumoniae* and those that were not (*p* = 0.022). There was also a significant difference in alpha diversity between children colonised with *H. influenzae* and those who were not (*p* = 0.017). No significant difference was found for SARS-CoV-2. *Sphingomonas*, *Ralstonia* and Rothia were significantly enriched in non-carriers of *S. pneumoniae*; *Actinobacillus* was significantly enriched in non-carriers of *H. influenzae*; *Actinobacillus* and *Porphyromonas* were significantly enriched in non-carriers of SARS-CoV-2 (*p* < 0.001).

**Discussion:**

Nearly 40% of children showed clinical symptoms of infection not related to geographical location. The OPM showed an imbalance during *H. influenzae* and *S. pneumoniae* carriage. This study provides a baseline understanding of microbiome markers in URTIs in children for future research, to develop targeted interventions aimed at restoring the microbial balance and reducing the symptoms associated with RTIs.

## 1 Introduction

The human microbiome, represented by 10 to 100 trillion symbiotic microbial cells, is considered beneficial to the human host; it is involved in the differentiation of mucosal structure and function, and stimulates innate and adaptive immune systems (Ursell et al., 2012). The composition of the pharyngeal microbiota and the collection of its genetic information, “microbiome”, plays a crucial role in maintenance of respiratory health by providing “colonization resistance” against pathogen invasion (Blaser and Falkow, 2009). However, an imbalance in its composition, for example through acquisition of new pathogens, viral co-infection, or other host or environmental factors, has been associated with increased risk of infections such as pneumonia, cystic fibrosis, colds, Covid-19, and meningitis (de Koff et al., 2016; Piters et al., 2016; Xiang et al., 2020; Diallo et al., 2023).

Respiratory tract infections (RTIs) are a major cause of morbidity and mortality worldwide, despite effective treatments with antibiotics and availability of certain vaccines. According to a recent World Health Organization (WHO) report, they are the leading cause of death among children and adults worldwide, and are responsible for around 4.25 million deaths worldwide every year (Mayor, 2010; Bogaert et al., 2011; World Health Organization, 2020).

In the inter-tropical zone of Côte d’Ivoire these RTIs are one of the ten leading causes of death among children and represent the second cause of morbidity after malaria according to the annual Health report of 2020, with a morbidity rate of 54.77‰ (Centers for Disease Control and Prevention, 2023). Although little studied, restoring the airway microbiome is a possible route to prevent RTIs and could become an additional intervention tool alongside antibiotics and vaccines (Cho and Blaser, 2012). In the airways of children, viruses such as rhinoviruses, influenza viruses, respiratory syncytial virus, adenoviruses and coronaviruses are the main causes of viral respiratory infections (Mahony, 2008). Bacteria such as *Haemophilus influenzae, Corynebacteria* species, *Streptococcus pneumoniae, Staphylococcus aureus, Moraxella catarrhalis and Prevotella melanino-genica* frequently cause bacterial respiratory infections (Brook et al., 1981; Karaman et al., 2009; Maleki et al., 2020).

Some of these potential pathogens are also commonly found as members of the normal flora of the human oral cavity and can be detected in nasal or respiratory secretions. The asymptomatic or symptomatic presence of these microorganisms, combined with a general lower application of hygiene measures, make children more likely to transmit these pathogens to others. The likelihood of transmission is further enhanced when large numbers of children are gathered, such as in day care centres and schools (Msd Manual Consumer Version, 2023). It is therefore important to characterize the composition of the OPM of schoolchildren in order to better prepare public health interventions. The concept of using microbial markers as a non-invasive diagnostic tool for diseases has been gradually developed due to new sequencing technologies (Gao et al., 2021). The presence of microorganisms, and their associated metabolites, can provide signatures specific to an infection (Times, 2020). Microbial signatures are used, not for the diagnosis of infections as such, but to predict certain aspects of classical infectious diseases, such as disease severity and progression (Khanna et al., 2016). Microbiome can be affected by multiple factors, such as life style, diet, environment and therefore studies in a particular setting may not be representative of the microbiome elsewhere. However, in Côte d’Ivoire, to our knowledge, there are no studies of the OPM in children and therefore no baseline data to build on.

A better understanding of the interactions between micro-organisms in the oropharynx will contribute to the development of new therapeutic agents or preventive measures likely to improve respiratory health outcomes and help stratify at-risk populations to better target current interventional approaches. This study aims to characterize the variations in the OPM of school children from northern and southern Côte d’Ivoire with visible clinical upper respiratory tract infections (URTI) in order to determine potential biomarkers that could be used for surveillance and responses.

## 2 Materials and methods

### 2.1 Study sites and participants

Two sites were selected for this study conducted in Côte d’Ivoire, West Africa: one in the northern town of Korhogo and one in the southern city of Abidjan. A public school was targeted in both cities. Children between 8 and 12 years old were invited to participate in the study that was conducted from November 2020 to April 2021. A cohort of schoolchildren was selected and followed in two primary school in Korhogo (*n* = 37) and Abidjan (*n* = 39). These numbers derived from published studies of the nasopharyngeal microbiome that used a similar sample size and were able to identify a distinct microbial profile and relevant changes (Biesbroek et al., 2014; Borges et al., 2018). The inclusion factors were school enrolment, age between 08 to 12 years and consent from parents. Children outside this age range were excluded.

That cohort was skewed toward girls, with a total of 29 girls (76.3) and 9 boys (23.7) in Abidjan. Korhogo had a more balanced male-female ratio, with 19 girls (51.4%) and 18 boys (48.6%).

Prior to inclusion in the study, informed consent was obtained from parents or legal guardian and written assent from the participant older than 10 years. Ethical approval from the National Ethical Committee was also obtained (IRB000111917). A questionnaire on infection risk factors and oral health was also administered to participants (Supplementary Tables 1, 2) by the team medical doctor.

Oral health questionnaire was completed by the team doctor every month prior to sampling. The criteria for consideration as symptomatic URTIs were the presence of visible signs such as nasal discharge, swelling, redness, irritation of the throat.

The risk factor questionnaire was completed by children’s parents or legal guardians after obtention of the signed informed consent. Nutritional status was defined in terms of the child’s body mass index. The modalities considered for the analysis were: Mild malnutrition, Moderate malnutrition, Severe malnutrition, normal, obesity and excess weight. A nurse from the school’s associated clinic, part of the health system in Côte d’Ivoire, was also present at each visit to attend to the children care and refer them, to the clinic if deemed necessary. Children aged from 8 to 12 years were invited to participate based on the school administration list, using a random selection to obtain the total number of participants. One participant dropped out of the study in Abidjan (S2), the other variation in number of participants were due to children absence from school on respective sampling days.

### 2.2 Sample collection

Oropharyngeal swab samples were collected monthly from each participant using sterile swabs. Swabs were stored in 1 ml of RNAprotect to protect RNA stability. RNAprotect samples were aliquoted into two different tubes and stored at −80°C. The first sample was used for 16S rRNA sequencing and RT-PCR detection of *S. pneumoniae* and *H. influenzae*. The second one was used for detection of SARS-CoV-2.

All swab samples were collected by qualified medical personnel trained in the study’s sampling procedures under the supervision of school medical officers. The physician performed a clinical examination of the children’s oral health prior to any sampling to determine the presence of infections or visible irritations in the children’s throats. This made it possible to identify a number of children with visible sign of URTIs throughout the cohort (nasal discharge, swelling, redness and irritation of the throat); as well as children with no clinical signs of infection.

### 2.3 DNA extraction

DNA samples were extracted from the oropharyngeal samples using a Qiagen DNeasy PowerSoil kit (Qiagen, Germany) following the manufacturer’s instructions. DNA samples were quantified by a Qubit 4 fluorometer (Invitrogen, Carlsbad, CA, USA), and molecular size was estimated by 1% agarose gel electrophoresis.

### 2.4 PCR amplification of the V3-V4 region

The V3-V4 hypervariable region of the 16S rRNA gene was targeted for sequencing. Forward and reverse primers targeting this region were generated with an Illumina adapter overhang sequence appended to the primer pair for compatibility with Illumina index and sequencing adapters (Klindworth et al., 2013). Amplifications were done in 25 μl reactions with 12.5 μl Q5^®^ Hot Start High-Fidelity 2X Master Mix (NEB), 5 μl of 1 μM forward and reverse 16S primer and 2.5 μl of template. Reactions were carried out on ABI Veriti thermocyclers (Applied Biosytems) under the following conditions: 95°C for 3 min, 25 cycles of; 95°C for 30 s, 55°C for 30 s, 72°C for 30 s, followed by 72°C for 5 min and a final hold at 4°C. The amplified products were then verified on 1.5 % agarose gel with a product of ∼550 bp expected.

### 2.5 Library preparation and sequencing

Amplified products were further purified using Agencourt AMPure XP beads (BeckmanCoulter) for library preparation. Libraries were then prepared by ligating Illumina dual indices and Illumina sequencing adapters to the purified amplicons using the NexteraXT index kit. Attachment of the indices was performed using 5 μL of the 16S amplicon DNA, 5 μL of Illumina Nextera XT Index Primer 1 (N7xx), 5 μL of Nextera XT Index Primer 2 (S5xx), 25 μL of Q5^®^ Hot Start High-Fidelity 2X Master Mix (NEB), and 10 μL of PCR-grade water (Ambion). The reactions were carried out on ABI Veriti thermocyclers (Applied Biosytems) under the following conditions 95 °C for3 min, followed by 8 cycles of 95°C for 30 s, 55°C for 30 s, and 72°C for 30 s, a final extension at 72°C for 5 min and a final hold at 4°C. The libraries were then purified using Agencourt AMPure XP beads (BeckmanCoulter) and thereafter size distribution and library quality control performed using the Agilent 2100 Bioanalyzer (Agilent) to confirm the expected size distribution and the quality. The libraries were finally quantified using the Qubit dsDNA HS kit on the Qubit 4.0 flourometer (Life Technologies) normalized and pooled at equimolar concentration based on the Qubit results. A total of 10pM of the pooled library was then spiked with 8% Phix (v3) for sequencing. Sequencing was done on the Illumina MiSeq system using 2 x 300 bp PE sequencing with the MiSeq^®^ Reagent Kit v3 (600 cycle).

### 2.6 Identification of pathogens by RT-PCR

All the RT-PCR tests were done retrospectively on stored samples multiple months after the end of the sample collection.

• Bacterial RT-PCR

Detection of *Streptococcus pneumoniae* and *Haemophilus influenzae* was done using a multiplex assay targeting *S. pneumoniae*, *H. influenzae* and *Neisseria meningitidis*. A mastermix of 15 μL was prepared as follow: 7.5 μL of 2x Master Mix, 0.5 μL of each primer and probe, and 1 μL of MgCl2 along with 2 μL of DNA previously extracted for the 16SrNA sequencing. Primers and probes used are in Table 1.

**TABLE 1:** List of primers and probes used for bacterial RT-PCR.

Organisms	Primers and probes	Sequences (5′-3′)	5′ dye	3′ quencher	References
*S. pneumoniae*	*lytA*- CDC-F	ACGCAATCTAGCAGATGAAGCA			Carvalho et al., 2007
	*lytA-*CDC-R	TCGTGCGTTTTAATTCCAGCT			
	*lytA*- CDC-Probe	TGCCGAAAACGCTTGATACAGGGAG	ROX	BHQ2	
	*SP_2020_*F	TAAACAGTTTGCCTGTAGTCG			Tavares et al., 2019
	*SP_2020*_R	CCCGGATATCTCTTTCTGGA			
	*SP_2020_*Probe	AACCTTTGTTCTCTCTCGTGGCAGCTCAA	Cy5	BHQ2	
*H. influenzae*	*HelS*-F	CCGGGTGCGGTAGAATTTAATAA			Arjarquah et al., 2022
	*HelA*-R	CTGATTTTTCAGTGCTGTCTTTGC			
	*Hel*- Probe	ACAGCCACAACGGTAAAGTGTTCTACG	FAM	BHQ1	

### 2.7 RNA extraction

Ribonucleic acid (RNA) extraction was performed on oropharyngeal samples using the QIAamp viral RNA kit for viral pathogens (Qiagen, Germany) according to the manufacturer’s recommendations. RNA quality was checked using the Qubit RNA HS kit.

• **RT-qPCR for SARS-CoV-2**

RT-qPCR of SARS-CoV-2 was performed in 20 μL of reaction medium containing 10 μL of Luna Universal Probe One-Step Reaction Mix (2X), 1 μL of Luna WarmStart RT Enzyme Mix (20X), 0.8 μL of each E Sarbeco Forward and reverse primer (10 μM), 0.4 μL of E Serbeco Probe, 5 μL of Nuclease-free Water, and 2 μL of RNA. Primers and probe used have shown in Table 2 (Corman et al., 2020).

**TABLE 2:** Primers and probe used to target the SARS-CoV-2 E gene in oropharyngeal samples.

Primer/probe	Sequences (5′-3′)
E_Sarbeco_F1	ACAGGTACGTTAATAGTTAATAGCGT
E_Sarbeco_R2	ATATTGCAGCAGTACGCACACA
E_Sarbeco_P1	FAM-ACACTAGCCATCCTTACTGCGCTTCG-BBQ

### 2.8 16S data processing

The raw 16S rRNA sequences were analyzed using R-4.2.1 software. They were quality filtered and denoised using the filterAndTrim function of DADA2 (Divisive Amplicon Denoising Algorithm) (Callahan et al., 2016). Poor quality ends were truncated, with a truncation length of 270 nts and 260 nts for forward and reverse reads respectively. The resulting sequences were then dereplicated, denoised and merged, and the removeBimeraDenovo function was used to remove chimeras in these sequences. The resulting amplicon sequence variants (ASVs) were classified by taxonomy and mapped to a reference set of operational taxonomic units (OTUs) at 99% sequence similarity using the Silva database (Silva_nr_v138) (Quast et al., 2013) with the assign-Taxonomy function. The data set was decontaminated using the “decontam” package (Nm et al., 2018) and then combined into a phyloseq object.

### 2.9 Statistical analysis

Shannon index, alpha-diversity and relative abundances (at phylum and genus level) were performed using the phyloseq package (McMurdie and Holmes, 2013). Prior to statistical analysis, the data were rarefied with a depth of 30000 reads per sample. Shapiro-Wilk test were used to test data normality for quantitative variables before comparing microbial diversity between the groups.

Wilcoxon rank sum tests and Kruskal-Wallis were used to compare microbial diversity in participant groups. Fisher or Chi-square tests were used for categorical data from the risk factors questionnaire, as well as the relationship between clinical symptom of infections and geographical location. Values of *p* < 0.05 were considered significant. All data were plotted using the ggplot2 package.

Principal coordinate analysis (PCoA) was performed according to the Bray-Curtis dissimilarity matrix for beta diversity. The association between oropharyngeal microbiome and symptomatic URTIs was also tested by permutational multivariate analysis of variance using the vegan package.

Bacteria genus differentially enriched according to status (COVID-19 and not; *H. influenzae* and not; *S. pneumoniae* and not) were analyzed at OTUs level using the DESeq2 method (McMurdie and Holmes, 2013). The package DESeq2 provides methods to test bacteria differentially enriched by use of negative binomial generalized linear models.

## 3 Results

### 3.1 Clinical and RT-PCR diagnosis of upper respiratory tract infections

During the study, 17 (43.59%) participants in Abidjan and 15 (40.54%) in Korhogo had visible signs of URTI following the clinical examination, with 26 episodes of infection in Abidjan and 24 in Korhogo. Eleven children had repeated episodes of infection.

The prevalence of these symptoms by site, visit and sex is presented in Table 3. These symptoms of infection (runny nose, coughing, redness and throat irritation) were not differently associated with geographical location (*p* > 0.05; Fisher test). The change in prevalence of clinical symptoms of respiratory infections was significant throughout the different visits in the north (*p* = 0.026, Fisher test), but not in the south (*p* > 0.05), with a high prevalence in February and April. There was no significant difference between the application of hand hygiene measures, the nutritional status of participants (in terms of the child’s body mass index), promiscuity or contact with animals in the two study sites (*p* > 0.5). We also found no link between socio-economic factors and clinical symptoms of URT infections (*p* > 0.5).

**TABLE 3:** Prevalence of suspected clinical infections in both study sites.

	Visits						Gender		Total
Site	S1	S2	S3	S4	S5	S6	Boys	Girls	Episodes of infection
**Abidjan total (n)**	**38**	**38**	**38**	**38**	**37**	**36**	**53**	**172**	
Infection Yes n (%)	3 (7.9)	10 (26.3)	3 (7.9)	2 (5.26)	4 (10.81)	4 (11.11)	1 (1.89)	25 (14.5)	26
Infection No n (%)	35 (92.1)	28 (73.7)	35 (92.1)	36 (94.7)	33 (89.2)	32 (88.9)	52 (98.1)	147 (85.5)	199
**Korhogo total (n)**	**37**	**37**	**37**	**37**	**36**	**36**	**107**	**113**	
Infection Yes n (%)	1 (2.7)	3 (8.11)	1 (2.7)	8 (21.62)	4 (11.11)	7 (19.44)	7 (6.6)	17 (15)	24
Infection No n (%)	36 (97.3)	34 (91.9)	36 (97.3)	29 (78.4)	32 (88.9)	29 (80.6)	100 (93.4)	96 (85)	196

For visits, S represents the different surveys (S1=Survey 1, S6=Survey 6).

In addition to the clinical signs of infection identified, the proportions of micro-organisms targeted per site, visit and gender are shown in Table 4. *Haemophilus influenzae* (12%), *Streptococcus pneumoniae* (6%) and SARS-CoV-2 (6%) were identified in participants presenting symptoms of URTIs (Table 5). Co-infection between *H. influenzae* and *S. pneumoniae* (2%) was also observed in some children with clinical suspicion of URTI. The prevalence of *H. influenzae* and *S. pneumoniae* was associated with geographical location (*p* < 0.001; Chi-square tests) with a higher prevalence in Korhogo than in Abidjan.

**TABLE 4:** RT-PCR identification of *H. influenzae*, *S. pneumoniae*, and SARS-CoV-2 by site, visit and gender.

	Visits							Gender			
Site	S1	S2	S3	S4	S5	S6	*p*-value	Boys	Girls	Total episodes of infection	*p*-value
**Total Abidjan (n)**	**38**	**38**	**38**	**38**	**37**	**36**		**53**	**172**		
*H. influenzae* Yes n (%)	4 (10.5)	5 (13.16)	5 (13.16)	3 (7.89)	7 (18.92)	2 (5.56)	0.57	8 (15.09%)	18 (10.47%)	26	0.5
*H. influenzae* No n (%)	34 (89.5)	33 (86.84)	33 (86.84)	35 (92.1)	30 (81.08)	34 (94.44)		45 (84.91%)	154 (89.53%)	199	
*S. pneumoniae* Yes n (%)	0	3 (7.89)	1 (2.63)	0	1 (2.70)	1 (2.78)	0.32	3 (5.66%)	3 (1.74%)	6	0.14
*S. pneumoniae* No n (%)	38 (100)	35 (92.11)	37 (97.37)	38 (100)	36 (97.30)	35 (97.22)		50 (94.34%)	169 (98.26%)	219	
SARS-CoV-2 Yes n (%)	0	0	0	1 (2.63)	1 (2.70)	3 (8.33)	0.06	1 (1.89%)	4 (2.33%)	5	1
SARS-CoV-2 No n (%)	38 (100)	38 (100)	38 (100)	37 (97.37)	36 (97.30)	33 (91.67)		52 (98.11%)	168 (97.67%)	220	
**Total Korhogo (n)**	**37**	**37**	**37**	**37**	**36**	**36**		**107**	**113**		
*H. influenzae* Yes n (%)	5 (13.51)	7 (18.92)	9 (24.32)	12 (32.43)	11 (30.56)	12 (33.33)	0.28	31 (28.97%)	25 (22.12%)	56	0.31
*H. influenzae* No n (%)	32 (86.49)	30 (81.08)	28 (75.68)	25 (67.57)	25 (69.44)	24 (66.67)		76 (71.03%)	88 (77.88%)	164	
*S. pneumoniae* Yes n (%)	6 (16.2)	2 (5.41)	4 (10.81)	13 (35.14)	3 (8.33)	8 (22.22)	0.006	22 (20.56%)	14 (12.39%)	36	0.15
*S. pneumoniae* No n (%)	31 (83.8)	35 (94.59)	33 (89.19)	24 (64.86)	33 (91.67)	28 (77.78)		85 (79.44%)	99 (87.61%)	184	
SARS-CoV-2 Yes n (%)	0	0	0	7 (18.92)	3 (8.33)	0	<0.001	5 (4.7%)	5 (4.42%)	10	1
SARS-CoV-2 No n (%)	37 (100)	37 (100)	37 (100)	30 (81.08)	33 (91.67)	36 (100)		102 (95.3%)	108 (95.58%)	210	

“Yes” and “No” indicate the presence or absence of microorganisms. For visits, S represents the different surveys (S1 = Survey 1, S6 = Survey 6).

**TABLE 5:** Prevalence of microorganisms identified by RT-PCR.

		*H. influenzae*		*p*-value	SARS-CoV-2		*p*-value	*S. pneumoniae*		*p*-value	*H. influenzae* and *S. pneumoniae*		*P*-value
		Yes	No		Yes	No		Yes	No		Yes	No	
Clinical infections	Yes n (%)	**6 (12%)**	44 (88%)	0.25	**3 (6%)**	47 (94%)	0.23	**3 (6%)**	47 (94%)	0.6	**1 (2%)**	49 (98%)	0.34
	No n (%)	76 (19.24%)	319 (80.76%)		12 (3%)	383 (97%)		39 (9.9%)	356 (90.1%)		26 (6.58%)	369 (93.42%)	

Values in bold represent the prevalence of participants with symptoms of clinical infection and carrying microorganisms detected by RT-PCR.

### 3.2 Characterization of the structure of the oropharyngeal microbiome based on 16S rRNA analysis

**FIGURE 1:**
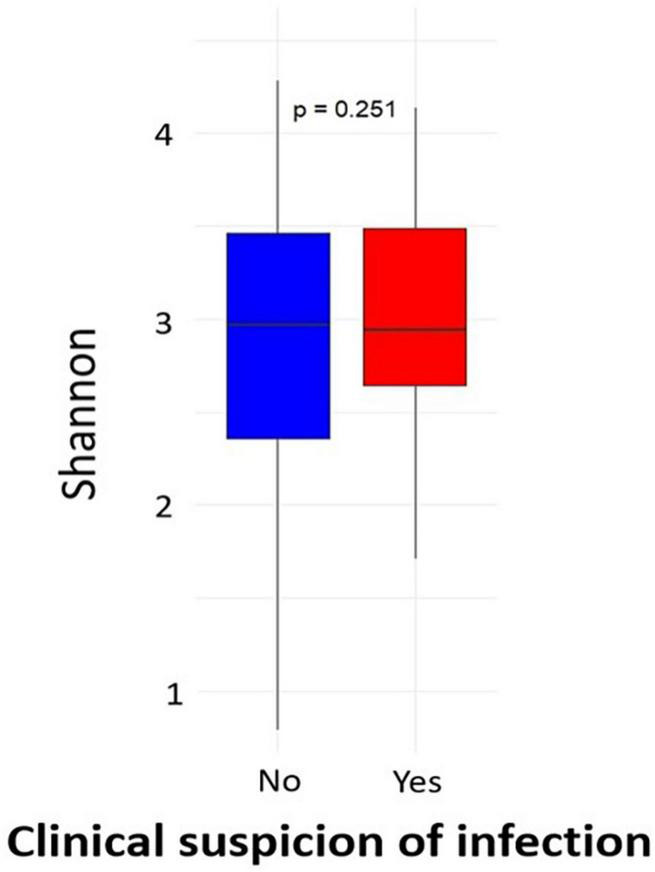
Alpha diversity (Shannon index) of the oropharyngeal microbiome in participants with clinical symptom of infections and healthy children.

**FIGURE 2:**
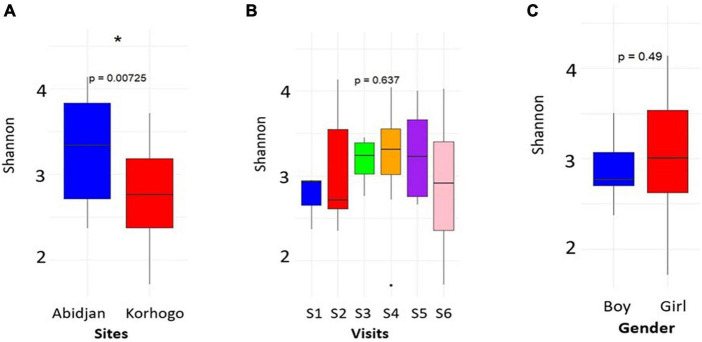
Plots showing the alpha diversity (Shannon index) of the oropharyngeal microbiome in participants by site **(A)**, visit **(B)** and gender **(C)**. The asterisk shows a geographical difference in the participants’ microbiome. For visits, S represents the different surveys (S1 = Survey 1).

Of the 434 samples received after sequencing, 427 passed the filter for 16S rRNA analysis. Of these sequences, 46 were from participants with clinical symptoms of infection out of 50. Analysis of alpha diversity showed that there was no significant difference between the microbial profile of children with clinical suspicion of upper respiratory infection and that of uninfected children using Shannon’s diversity index (*p* = 0.25) (Figure 1). A geographical difference was however observed in the microbial profile of children from the two study sites (Abidjan and Korhogo) showing a greater abundance and microbial diversity in individuals from Abidjan than in those from Korhogo using a Shannon diversity index (*p* < 0.001, Wilcoxon rank-sum test) (Figure 2). No significant difference was observed in the microbial profile of participants over time and according to gender (*p* > 0.5).

**FIGURE 3:**
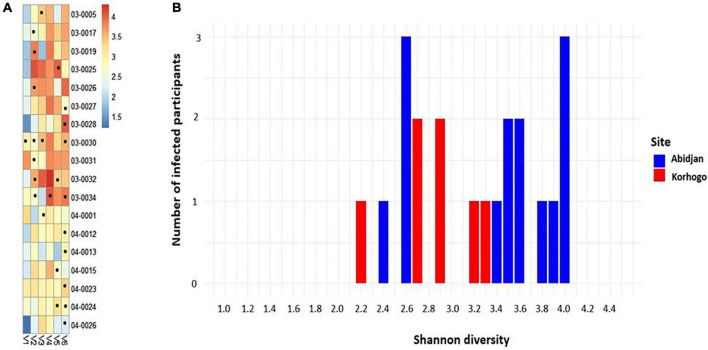
Heat map of the relative abundances of differential OTUs in the group of positive samples whose sequences were received. Each point represents the visit during which the participant had a symptom of irritation, redness or inflammation in the throat **(A)**. Bar plot showing the number of people with clinical infections as a function of Shannon diversity at each study site (blue in Abidjan and red in Korhogo) **(B)**. **(B)** Produced taking into account the Shannon diversity of the first figure.

Microbial diversity of each individual who had at least one URTI symptom during the cohort was plotted over time (per visit). This showed a generally high microbial abundance in the presence of infection. More people were infected when Shannon diversity was high in Abidjan, with values between 2.4 and 4 (Figure 3).

**FIGURE 4:**
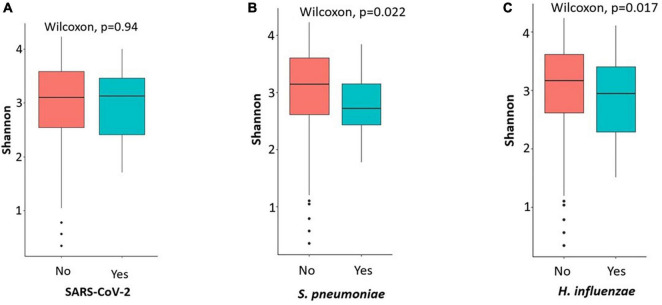
Plots showing the alpha diversity (Shannon index) of the oropharyngeal microbiome in participants carriers of SARS-CoV-2 and not **(A)**, *S. pneumoniae* **(B)** and *H. influenzae* **(C)**. Microbial diversity was low when participants were carriers of *H. influenzae* and *S. pneumoniae*.

Analysis of alpha diversity showed a significant difference between symptomatic carriers and non-carriers of *S. Pneumoniae* and *H. influenzae* (*p* < 0.05). Microbial diversity was lower in participants carrying both micro-organisms. Alpha diversity showed no association between symptomatic carriers and non-carriers of SARS-CoV-2 (*p* = 0.94) (Figure 4). Beta diversity results (Supplementary Figure 1) also revealed that symptomatic carriers and non-carriers of *S. Pneumoniae* and *H. influenzae* have distinct OPM compositions (*p* = 0.001).

**FIGURE 5:**
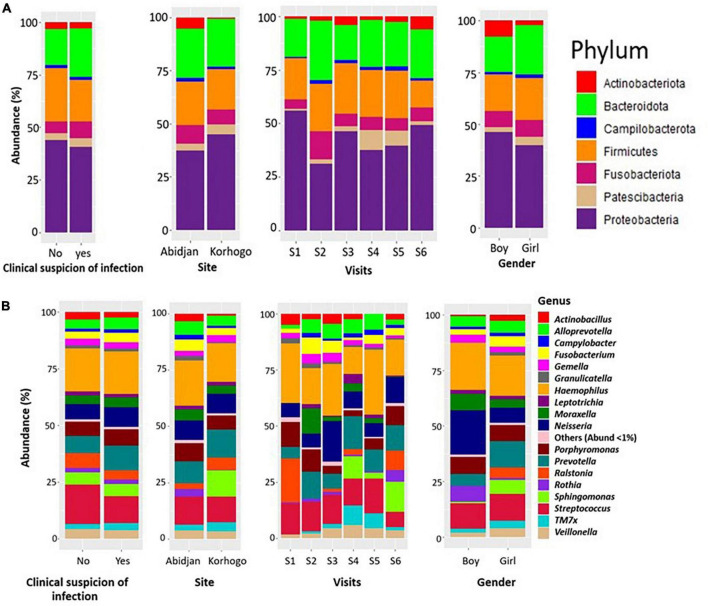
Bar charts illustrating the relative abundance of bacterial phylum **(A)** and genus **(B)** in participants with clinically suspected throat infection throughout the cohort. The representations were made generally, according to site, visit and gender.

The most represented phyla in the samples were Proteobacteria, Bacteroidota, Firmicutes and Fusobacteriota with proportions higher than 15%. At the genus level, 18 different genera were detected in the children’s oropharynx, with an abundance greater than 1%. Taxonomic classification also showed that the relative abundances of the different bacterial genera in the participants’ upper respiratory tracts differed from one infection to another. We found that the genus *Streptococcus* were significantly more abundant in children who had no clinical symptoms of infection than in those who did (17.37% VS 11.92%, *p* = 0.001) (Figure 5).

**FIGURE 6:**
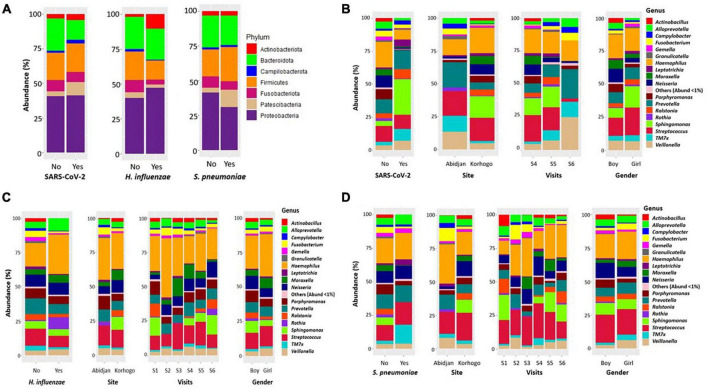
Bar charts illustrating the relative abundance of bacterial phylum **(A)** and genera in the oropharynx of participants carrying SARS-Cov-2 **(B)**, *H. influenzae* **(C)** and *S. pneumoniae* **(D)** All bacterial genera whose abundance was less than 1% were represented by “other.”.

When zooming only on children that had clinically diagnosed URTI, *Leptotrichia* was dominant in carriers of *S. pneumoniae* compared to non-carriers (4.27%; 1.45%; *p* = 0.028). The abundance of *Actinobacillus* was also very low in all three types of carriage (Figure 6). The proportions of these different genus in terms of abundance are shown in Table 6. Identification to species level was not possible for all ASVs.

**TABLE 6:** Relative abundance of the 18 most abundant bacterial genera in the microbiome of individuals according to whether or not they are symptomatic carriers.

Genus	Clinical infections		P	Sars-Cov-2			*H. influenzae*			*S. pneumoniae*		
	No	Yes		No	Yes	p	No	Yes	p	No	Yes	p
*Actinobacillus*	3.35	2.38	0.499	2.54	0	0.397	2.73	0	0.205	2.54	0	0.769
*Alloprevotella*	3.89	5.17	0.792	5.4	1.77	0.4	4.57	9.15	0.187	5.01	7.43	0.165
*Campylobacter*	1.42	1.67	0.202	1.58	3.07	0.509	1.86	0.45	0.064	1.69	1.42	0.539
*Fusobacterium*	3.24	4.25	0.03	4.36	2.64	0.339	4.67	1.47	0.074	4.45	1.36	0.211
*Gemella*	2.77	2.63	0.218	2.78	0.49	0.123	2.91	0.78	0.082	2.58	3.38	0.982
*Granulicatella*	1.26	1.27	0.425	1.3	0.88	0.391	1.42	0.26	0.24	1.36	0	0.297
*Haemophilus*	19.12	18.69	0.852	19.49	7.34	0.065	17.2	28.67	0.08	18.62	19.79	0.767
*Leptotrichia*	1.72	1.63	0.542	1.37	5.29	0.111	1.77	0.68	0.36	1.45	4.27	**0.028**
*Moraxella*	3.79	4.28	0.831	4.53	0.7	0.604	4.08	5.57	0.225	4.55	0.41	1
*Neisseria*	6.72	8.74	0.82	9.27	1.08	0.127	8.74	8.71	0.473	8.65	10.04	0.838
*Porphyromonas*	6.06	7.1	0.465	7.57	0.39	**0.01**	7.3	5.77	0.894	7.38	3.09	0.306
*Prevotella*	7.73	11	0.019	10.76	14.57	0.265	11.59	7.09	0.433	10.89	12.63	0.802
*Ralstonia*	6.47	3.96	0.196	3.74	7.06	0.191	4.19	2.41	0.559	4.23	0	0.28
*Rothia*	1.96	1.95	0.296	2.05	0.49	0.453	0.91	8.85	0.749	2.08	0	0.1
*Sphingomonas*	5.42	5.34	0.636	3.84	26.83	0.095	5.44	4.69	0.985	5.71	0	0.192
*Streptococcus*	17.37	11.92	**0.001**	12.01	10.71	0.847	12.47	8.28	0.286	11.59	16.77	0.09
*TM7x*	2.32	3.2	0.555	2.79	8.97	0.057	3.44	1.59	0.534	2.44	14.05	0.371
*Veillonella*	4.14	3.65	0.167	3.41	7.08	0.4	3.53	4.44	0.432	3.64	3.85	0.601
*Others*	1.24	1.17		1.21	0.64		1.18	1.14		1.15	1.53	

The different p-values were calculated using the Wilcoxon rank sum test.

### 3.3 Determination of microbial markers involved in symptomatic upper respiratory tract infections

Microbial markers were identified as predominant in carriage of each microorganism. The results revealed that the genera *Sphingomonas, Ralstonia* and *Rothia* were significantly enriched in the absence of *S. pneumoniae* (5.71%; 4.23%; 2.08%) when compared to carriers (0%) (*p* < 0.001; log2 fold-change: −26.1; −24.8; −9.9). *Actinobacillus* was significantly enriched in the absence of *Haemophilus influenzae* (2.73% VS 0%; *p* < 0.001); *Actinobacillus* and *Porphyromonas* were microbial markers of presence of SARS-CoV-2 (0%; 0.39%) when compared to non-carriers (2.54%, 7.57%; with *p* < 0.001; log2 fold-change: −25.8 and −5.1) (Table 7).

**TABLE 7:** Table showing the results for the most significant genera and phyla in the three types of infections using the DESeq2 differential abundance analysis.

*H. influenzae* carriage											
**baseMean**	**log2Fold Change**	**lfcSE**	**stat**	**pvalue**	**padj**	**Kingdom**	**Phylum**	**Class**	**Order**	**Family**	**Genus**
449.478154	−26.08070999	3.73333343	−6.9859043	2.83027E−12	5.09449E−11	Bacteria	Proteobacteria	Gammaproteobacteria	Pasteurellales	Pasteurellaceae	*Actinobacillus*
***S*.*pneumoniae* carriage**											
880.016161	−26.12988877	4.16702087	−6.2706402	3.59567E−10	6.4722E−09	Bacteria	Proteobacteria	Alphaproteobacteria	Sphingomonadales	Sphingomonadaceae	*Sphingomonas*
966.564475	−24.75841785	4.52357889	−5.4731925	4.42E−08	3.978E−07	Bacteria	Proteobacteria	Gammaproteobacteria	Burkholderiales	Burkholderiaceae	*Ralstonia*
181.960938	−9.963195029	2.71187828	−3.6739094	0.000238867	0.001433205	Bacteria	Actinobacteriota	Actinobacteria	Micrococcales	Micrococcaceae	*Rothia*
**Sars-Cov-2 carriage**											
449.478154	−25.78980787	5.3122002	−4.854826	1.20492E−06	1.92788E−05	Bacteria	Proteobacteria	Gammaproteobacteria	Pasteurellales	Pasteurellaceae	*Actinobacillus*
1721.68874	−5.128442329	1.11016396	−4.619536	3.84599E−06	3.07679E−05	Bacteria	Bacteroidota	Bacteroidia	Bacteroidales	Porphyromonadaceae	*Porphyromonas*

## 4 Discussion

The importance of respiratory infections in children, particularly in low- and middle-income countries, is very evident as it has been shown that 30% of the annual mortality rate in children is associated with acute respiratory infections (Liu et al., 2018). This study is the first to report on the oropharyngeal microbiome in children with suspicions of URTIs in Côte d’Ivoire using 16S rRNA sequencing.

Several participants were attending school despite harboring symptoms of respiratory tract infections such as nasal discharge, swelling, redness and irritation of the throat. This suggests that common URTIs symptoms (runny nose, coughing, etc.) are not considered alarming enough for some parents to keep children at home. Previous research has shown that infectious disease management in schools is essential to minimize the spread of respiratory infections (Ridenhour et al., 2011). In addition, health campaigns promoting hand hygiene and use of hand sanitiser have been shown to be effective in reducing illness and absenteeism (White et al., 2005; Azor-Martínez et al., 2014).

Variation in clinical symptoms of respiratory infections was significant throughout the year in participants in Korhogo (north) but not in Abidjan (south), with a high prevalence in February and April. This means that seasonality could be a factor to be taken into account in the occurrence of these infections in the northern part of the country, given that the different sampling waves covered both the dry season and the approach of the rainy season. Previous studies have shown a link between the seasonality of URTIs and environmental factors. In fact, certain viruses such as rhinovirus, adenovirus and influenza viruses A and B have been more active during certain seasons. Temperature and humidity also played a role in their transmission. (Price et al., 2019).

Paynter et al. (2015) have also shown that human immunity varies according to seasons, which could contribute to the seasonal nature of respiratory infections. This study also confirmed the presence of *Haemophilus influenzae, Streptococcus pneumoniae* and SARS-CoV-2 in the participants’ oropharynx. Despite the fact that these three types of infections were more strongly detected in children in the north than in those in the south, *Haemophilus influenzae* remained the most common species carried in the oropharynx.

These results are in line with a previous study showing the persistence of *Haemophilus influenzae* as a major cause of acute respiratory infections in sub-Saharan Africa (Lagare et al., 2015).

The fact that the prevalence of carriage of these micro-organisms is considerably different from one area to another may be due to a number of factors, such as seasonal difference between the two sites and promiscuity (Wimalasena et al., 2021). The link between seasonality and respiratory infections has also been demonstrated in another study in Kenya that showed an association between the rainy season and nasopharyngeal carriage of *Streptococcus pneumoniae* and *Haemophilus influenzae* independent of the effect of age (Abdullahi et al., 2008).

The role of children in the transmission of COVID-19 has been a subject of debate worldwide (Dattner et al., 2021). A study by Jiehao et al. (2020) of children diagnosed with COVID-19 indicates that the potential risk of transmission from infected children to adult contacts should not be overlooked. Our study retrospectively identified COVID-19 patients with what can be considered benign to mild symptoms. SARS-CoV-2 was identified in this study in 15 participants between February and March 2021 (05 in Abidjan and 10 in Korhogo), coinciding with a period of high virus spread in Côte d’Ivoire according to WHO (World Health Organization, 2024). This results reinforce the idea that children were mostly asymptomatic carriers during the pandemic and could have played a role in the transmission of the virus to their household members (Zhu et al., 2020). More carriage studies in African children could have helped better study the diversity of the virus.

16S rRNA sequencing analysis showed that the oropharyngeal microbial diversity was significantly reduced in children who were symptomatic carriers of *H. influenzae* and *S. pneumoniae* infection, indicating the presence of oropharyngeal microbial dysbiosis in these children. Recent data using whole genome sequencing have also shown a reduction in the diversity of the oropharyngeal microbiome in elderly and adult patients with *Streptococcus pneumoniae* associated pneumonia (Piters et al., 2016). Laufer et al. (2011) also observed lower microbial diversity in the presence of *S. pneumoniae* in the URT of children with otitis media. These results suggest that dysbiosis of the URT microbiome may be associated with various respiratory infections, underlining the importance of maintaining a healthy microbial balance in this region.

16S rRNA analysis also showed that the dynamics of the microbiome between children in the north and south differed significantly (*p* < 0.001); with greater bacterial diversity in children from Abidjan than those from Korhogo. This could be explained by different lifestyles, including diet, genetics and host physiology as demonstrated in a previous study conducted on the salivary microbiome of populations living in different geographical and climatic environments (Li et al., 2014). It shows that there is considerable geographic variation in the microbiota of selected individuals. This diversity should be taken into account in future control strategies based on microbiota modulation.

The phyla that were highly represented in both groups of participants (children with clinical suspicion of infections and not) are Firmicutes, Proteobacteria and Bacteroidota with relative abundance above 15%. These are part of the five major bacterial phyla identified by the Human Microbiome Project (ScienceDirect Topics, 2007; Gao et al., 2014). Other phyla such as Fusobacteriota and Actinobacteriota were also represented to a lesser extent in the microbiome of children in this cohort. The identification of most of these phyla was also done in the study by Xia et al. (2022) on the pulmonary microbiome. This could be explained by the fact that the lungs microbiota represents an extension of the upper respiratory tract. The oropharynx is the junction between the mouth, nasopharynx, larynx, lower respiratory tract and gastrointestinal tract. It is also exposed to exogenous and endogenous microorganisms (Chen et al., 2021). Therefore, the set of species in the oropharyngeal microbiota may generally be larger than in other niches.

Of the microbial genera most present in terms of proportion (*Haemophilus, Streptococcus, Prevotella, Neisseria and Porphyromonas*), only *Streptococcus* showed a statistically significant difference in abundance between the groups of symptomatic and non-symptomatic children (*p* = 0.001). The prevalence of these bacteria in the oropharyngeal microbiome has been demonstrated in previous studies in healthy and asthmatic children, those suffering from Cystic Fibrosis, and in studies focusing on COVID-19 (Charlson et al., 2011; Boutin et al., 2015; Depner et al., 2017).

The genus *Sphingomonas, Ralstonia* and *Rothia* were identified as microbial markers of symptomatic carriage of *S. pneumoniae*. This suggests that their low abundance may also play a role in the modification of the microbiome and the onset of infection, as these three bacterial genera were not present in infected children. These results differ from those of Piters et al. (2016) who showed a significantly higher relative abundance of *Rothia* in oropharyngeal microbiota of elderly pneumonia patients, suggesting that *Rothia* may play a role in the pathogenesis of the infection. *Rothia* has also been implicated in T helper 17 (Th17)-induced lung inflammation and pneumonia in immunocompromised patients (Segal et al., 2016). Furthermore, abundance of this microorganism in the upper respiratory tract has also been associated with an increased risk of otitis media in children (Laufer et al., 2011), suggesting a potential role for this bacterium in the pathogenesis of respiratory infections generally. Thus, variations in microbial composition between different age groups could contribute to differences in susceptibility to infection (Belibasakis, 2018). *Ralstonia*, in particular the species *insidiosa*, has been isolated from the respiratory tract of cystic fibrosis patients (Lin et al., 2023). This bacterium has low toxicity and is a conditional pathogen. However, contraction of bacterial or viral pathogens, environmental factors or immunological disturbances can potentially lead to dysbiosis and proliferation of pathogens, which could result in symptomatic infections due to this microorganism.

The genus *Actinobacilus* and *Porphyromonas* were identified as microbial markers in symptomatic SARS-CoV-2 carriage with very low abundance in carriers’ individuals (*p* < 0.001). This decrease in *Actinobacillus* genus and *Porphyromonas* was observed in several previous studies in patients with COVID-19 (Ren et al., 2021; Soffritti et al., 2021; Wu et al., 2021; Cui et al., 2022; Gupta et al., 2022; Rafiqul Islam et al., 2022). These results suggest a potential role for these bacteria in the pathogenesis of COVID-19. However, further research is needed to fully understand the impact of these microbial markers in the context of COVID-19. *Actinobacilus* has also been identified as a microbial marker in symptomatic *Haemophilus influenzae* carriers.

These results demonstrate the importance of conducting regular surveillance of the carriage of upper respiratory tract pathogens in order to identify bacteria or viruses that are most prevalent and to prepare for the deployment of appropriate public health measures in the event of an epidemic. The microbiome results presented could be used as baseline data for the identification of biomarkers involved in URTIs in children despite the limited number of individuals.

The human upper respiratory tract microbiome is being studied more and more thanks to the development of new sequencing tools. However, the oropharynx has received less attention and, to our knowledge, few studies have been performed on the microbiome in Africa, especially on diseases of public health importance (Allali et al., 2021; Diallo et al., 2023). Additional studies including larger numbers of participants with a stratified spectrum of respiratory infections severity, will be useful to better understand the changes of the microbiota based on the progression of the disease.

The integration of other omics technologies such as RNA sequencing or metatranscriptomics will provide more specific gene expression information to better understand the metabolic characteristics of the children respiratory microbiome (Shakya et al., 2019). This approach could lead to a better understanding of the disease and more targeted approaches for treatment and prevention.

This study has certain limitations. Firstly, it used a cohort and therefore generalization of the results must be done with caution. Secondly, we were unable to identify causal relationships between symptomatic carriage of microorganisms and microbial markers in the oropharynx using either functional or mechanistic data as this would require whole genome metagenomic. Finally, the monthly sampling may have prevented the identification of rapidly evolving changes in the microbiome focusing on rather long-lasting changes.

## 5 Conclusion

In our study cohort 40% of children showed clinical symptoms of infection without geographical location variation.

OPM of children is highly diverse and varies considerably between both sites with greater microbiota diversity in Abidjan (stable seasonality humid) than in Korogho (variable seasonality dry/rainy) in children presenting clinical signs of URT infection. A significant difference in the microbiota diversity was also found in children that carry *S. Pneumoniae* and *H. influenzae* (*P* < 0.05) with lower microbial diversity in patients with both infections. However, the OPM was not significantly different when comparing children with or without SARS-CoV-2 carriage. 16S rRNA analysis showed that *Sphingomonas, Ralstonia and Rothia* were significantly enriched by non-carriers of *S. pneumoniae; Actinobacillus* was significantly enriched by non-carriers of *H. influenzae; Actinobacillus* and *Porphyromonas* were significantly enriched by non-carriers of SARS-CoV-2 (*p* < 0.001). Our study has shown that changes in the microbiome composition may favor predisposition for certain microorganisms to cause symptomatic infections. Further characterization of the response of the microbiota to airway infections down to the species level as well as gene expression using novel sequencing technologies such as shotgun sequencing or metatranscriptomics is needed to better understand the transcriptional changes of the host and microbiota during infection. This will provide a better understanding of pathogenesis of respiratory tract infections in children over the long term, and will help understand the effects of current and future preventive measures.

## Data availability statement

The datasets presented in this study can be found in online repositories. The names of the repository/repositories and accession number(s) can be found below: https://www.ncbi.nlm.nih.gov/sra/PRJNA1064912, from SRX23222127 to SRX23221695.

## Ethics statement

This project was approved by the Ethics Committee for Life Sciences and Health (CNESVS) of the Ministry of Health and Public Hygiene of the Republic of Côte d’Ivoire before the work was carried out; (Ethical approval number: IRB000111917). The National Programme for School and University Health (PNSSU) was associated to the study from the onset (selection of schools) and throughout the process to ensure the safety of the interventions. Written informed consent for participation in this study was provided by the participants’ legal guardians/next of kin.

## Author contributions

KM: Formal analysis, Investigation, Methodology, Visualization, Writing – original draft, Conceptualization, Validation. KD: Conceptualization, Funding acquisition, Investigation, Methodology, Project administration, Resources, Writing – original draft, Writing – review and editing, Formal analysis, Supervision, Validation. KB: Supervision, Validation, Writing – review and editing. KT: Investigation, Methodology, Writing – original draft, Validation. KG: Investigation, Methodology, Writing – review and editing. L-ST: Formal analysis, Methodology, Visualization, Writing – review and editing, Validation. AO: Formal analysis, Validation, Writing – review and editing, Visualization. BG: Supervision, Writing – review and editing. JN: Data curation, Methodology, Writing – review and editing, Validation. RW: Funding acquisition, Supervision, Writing – review and editing, Validation, Project administration. GA: Project administration, Supervision, Writing – review and editing, Resources, Validation. BB: Investigation, Project administration, Resources, Supervision, Validation, Writing – review and editing.
