# Supplementary material for: Association of symptomatic upper respiratory tract infections with the alteration of the oropharyngeal microbiome in a cohort of school children in Côte d’Ivoire
Source: Front Microbiol. 2024 Jun 27;15:1412923. doi: 10.3389/fmicb.2024.1412923 (PMC11238735; doi:10.3389/fmicb.2024.1412923)
Supplement: Supplementary file 1 [file Presentation_1.pdf]

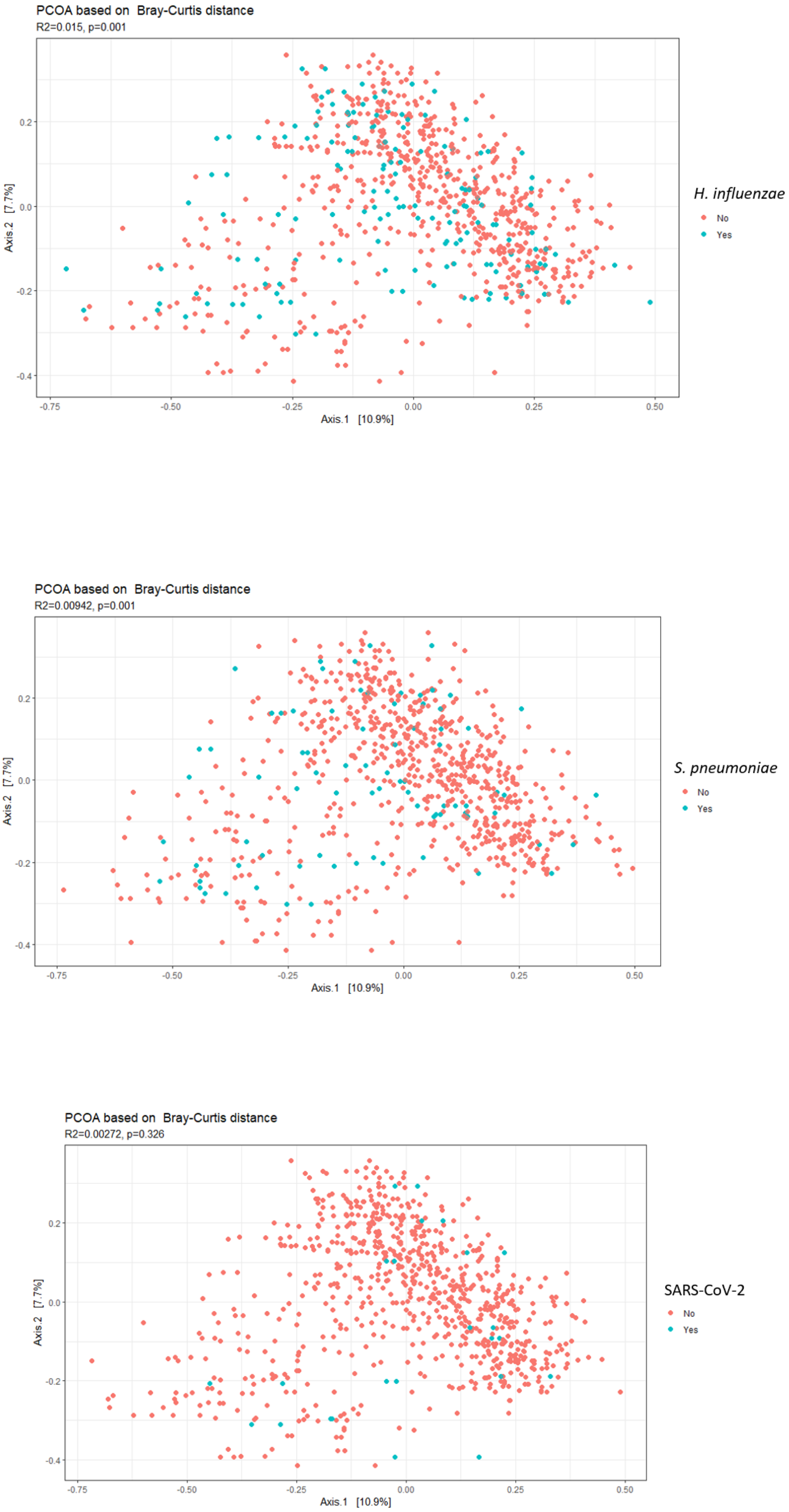

Supplemental figure 1: Beta diversity results showing the association between the oropharyngeal microbiome and symptomatic or non-symptomatic carriage of the three targeted microorganisms.
