# Supplementary material for: Association of symptomatic upper respiratory tract infections with the alteration of the oropharyngeal microbiome in a cohort of school children in Côte d’Ivoire
Source: Front Microbiol. 2024 Jun 27;15:1412923. doi: 10.3389/fmicb.2024.1412923 (PMC11238735; doi:10.3389/fmicb.2024.1412923)
Supplement: Supplementary file 2 [file Data_Sheet_1.pdf]

Supplemental table 1: Questionnaire on risk factors

**Question #**	**Question of identification**	**Response**
**1.**	Study ID	0 3-_ _ _ _
**2.**	Date of administration of the questionnaire [D/M/Y]	_ _/_ _/_ _ _ _
**3.**	The gender of the participant	Girl Boy
**4.**	Date of birth [D/M/Y]	_ _/_ _/_ _ _ _
	**Socio-economic aspect**	
**5.**	How many people live in your house?	#
**6.**	How many bedrooms are there in your house?	#
**7.**	How many toilets are there in your house?	#
**8.**	Where is the toilet in your house?	Indoor Outdoor
**9.**	Are there smokers in your house?	No Yes #
**10.**	Where is the kitchen in your home?	Indoor Outdoor
**11**	What fuel do you use for cooking?<br>*More than one answer can be circled*	Gas<br>Electricity<br>Paraffin<br>Coal<br>Wood<br>Straw<br>Cow dung
**12.**	How do you get to school and back home?	Personal car<br>Personal motorbike<br>Public transport (Taxi, gbaka, wôrô wôrô...)<br>I walk
**13.**	What is your father's job?	Primary sector (Agriculture, Livestock)<br>Secondary Sector (Industries)<br>Tertiary sector (Service)<br>No employment
**13.b**	What is your father's level of education?	Primary<br>Secondary<br>High School<br>University<br>No study

**14.**	What is your mother's job	Primary sector (Agriculture, Livestock)<br>Secondary Sector (Industries)<br>Tertiary sector (Service)<br>No employment
**14.b**	What is your mother's level of education?	Primary<br>Secondary<br>High School<br>University<br>No study
	**Environmental aspects**	
**15.**	Do you have animals at home?	No Yes
**15.a**	If yes, which ones?	Dog Cat Chicken<br>Sheep Goat Cow<br>Horse Other: ____________
**15.b**	Do your pets come inside the house?	No Yes
**15.c**	Where are these animals kept?	No Enclosure<br>Park Other: ....
**15.d**	Who are the family members who look after these animals?	The children the father<br>The mother Everyone
**16.**	Do you consume cow's milk at home?	Yes No
**16.b**	If yes, is it boiled (pasteurised)	Yes No
	**Health and Nutrition**	
**17.**	Do you know about meningitis?	Yes No
**17.b**	If yes, do you fear this disease?	Yes No
**17.c**	If yes, do you think it is a fatal disease?	Yes No
	If yes, do you think it is a contagious disease?	Yes No
**18**	Have you been vaccinated against meningitis?	Yes No
**18.a**	Do you have your vaccination record?	Yes No
**18.b**	Can the answer to question **18** be confirmed?<br>Which meningitis vaccine was taken?	No Yes<br>____________________________
**19.**	Weight in kg	
**20.**	Size in cm	
**21.**	Forearm circumference measurement in cm	
**22.**	What meal do you eat every day?	Breakfast

		Lunch<br>Gouter<br>Dinner
**21.**	Do you wash your hands before every meal?	Always Often<br>Rarely Never

Supplemental table 2: Oral Health Questionnaire

	**Questions**	**Response**
**1.**	Study ID	0 3-_ _ _ _
**2.**	Date of administration of the questionnaire [D/M/Y]	_ _ /_ _ /_ _ _ _
**3.**	Have you had a sore throat since we last met?	No Yes
**4.**	Have you had a cough since we last met?	No Yes
**5.**	Have you had a runny nose since we last met?	No Yes
**6.**	Have you had another illness since we last met?	No Yes
**6.a**	If yes, which one?	____________________________
**7.**	Have you seen a doctor?	No Yes
**7.a**	Have you taken any medication?	No Yes
	If yes, which one?	____________________________
**8.**	Has anyone living in your house had a sore throat, cough or runny nose since we last met?	No Yes
**9.**	Are there visible sores or bleeding in the mouth *(Doctor's observation)*	No Yes
**10.**	Is the throat visibly irritated? *(Doctor's observation)*	No Yes
